# Predicting Outcome in Comatose Patients: The Role of EEG Reactivity to Quantifiable Electrical Stimuli

**DOI:** 10.1155/2016/8273716

**Published:** 2016-04-03

**Authors:** Gang Liu, Yingying Su, Yifei Liu, Mengdi Jiang, Yan Zhang, Yunzhou Zhang, Daiquan Gao

**Affiliations:** Department of Neurology, Xuanwu Hospital, Capital Medical University, Beijing 100053, China

## Abstract

*Objective*. To test the value of quantifiable electrical stimuli as a reliable method to assess electroencephalogram reactivity (EEG-R) for the early prognostication of outcome in comatose patients.* Methods*. EEG was recorded in consecutive adults in coma after cardiopulmonary resuscitation (CPR) or stroke. EEG-R to standard electrical stimuli was tested. Each patient received a 3-month follow-up by the Glasgow-Pittsburgh cerebral performance categories (CPC) or modified Rankin scale (mRS) score.* Results*. Twenty-two patients met the inclusion criteria. In the CPR group, 6 of 7 patients with EEG-R had good outcomes (positive predictive value (PPV), 85.7%) and 4 of 5 patients without EEG-R had poor outcomes (negative predictive value (NPV), 80%). The sensitivity and specificity were 85.7% and 80%, respectively. In the stroke group, 6 of 7 patients with EEG-R had good outcomes (PPV, 85.7%); all of the 3 patients without EEG-R had poor outcomes (NPV, 100%). The sensitivity and specificity were 100% and 75%, respectively. Of all patients, the presence of EEG-R showed 92.3% sensitivity, 77.7% specificity, 85.7% PPV, and 87.5% NPV.* Conclusion*. EEG-R to quantifiable electrical stimuli might be a good positive predictive factor for the prognosis of outcome in comatose patients after CPR or stroke.

## 1. Introduction

Prognostication in comatose patients continues to be a challenge due to an increased survival rate with the help of medical developments [1, 2]. Moreover, many survival patients cannot recover consciousness and remain in a vegetative state (VS) [3, 4]. Thus, the accurate prediction of outcome avoids futile medical treatment in patients with irreversible damage. However, clinical tests show variable accuracy. The accuracy of clinical parameters is limited in intubated and aphasic patients. Moreover, absence of brainstem reflexes to stimuli is often limited by therapeutic hypothermia (TH) or sedative drugs in predicting prognosis of comatose patients.

Electroencephalogram (EEG), one of the most informative neurophysiological techniques available in the neurocritical care unit (NCU), can be used as a bedside complement to clinical evaluation in comatose patients. Previous studies have focused on the prognostic value of EEG reactivity (EEG-R) in comatose patients. EEG reactivity is a positive predictive factor for assessing the outcome of comatose patients. The comatose patients with EEG-R are prone to have good outcomes [5–7]. Logi et al. used only EEG reactivity to predict recovery of consciousness in postacute severe brain injury, mainly traumatic brain injury, and subarachnoid haemorrhage patients, and they confirmed EEG reactivity was strongly related to the recovery of consciousness [7]. It is widely accepted that EEG-R might reflect the connection of cortical neurons and ascending reticular activating system (ARAS), which may be the underlying mechanism for prognostication [8–10].

The reactivity of the EEG background is defined as the presence of any clear change in amplitude or frequency following the application of an external stimulus. The ordinary external stimulation often includes auditory stimuli, somatosensory stimuli, and visual stimuli [11, 12]. However, these external stimuli are often difficult to quantify in clinical practice. As a result, these difficulties in individually determining the accurate intensity and duration of stimulation might lead to deviations and decrease the prognostic value of EEG-R. This might explain the discrepancy among different studies that use the same stimulation method. Moreover, the thermal stimulus, a type of quantifiable stimulation, has achieved better prognostic accuracy for patients in VS or minimally conscious state (MCS) in a recent study [13].

Moreover, prognostic neurophysiological studies in comatose patients are still limited. Considering the fact that cardiopulmonary resuscitation (CPR) and stroke are two main causes of coma in NCU, we applied an electrical stimulation paradigm in comatose patients after CPR or stroke.

The aim of this study was to assess the value of the new quantifiable stimulation as a complementary tool in predicting neurological outcomes of adult comatose patients.

## 2. Methods

### 2.1. Patients

We prospectively enrolled all consecutive comatose adults after CPR or stroke. They were admitted to the Department of NCU in Xuanwu Hospital, Capital Medical University, between April 2014 and April 2015. The study was approved by the ethics committee of Xuanwu Hospital, Capital Medical University, and it adhered to the Declaration of Helsinki. Patients' relatives were asked for informed consent.

All patients were older than 18 years and unconscious, with a Glasgow coma score (GCS) below or equal to 8. Exclusion criteria were as follows: a known history of severe neurological deficit, terminal disease or expected lifespan of less than 3 months, spinal cord injury, and patients lost during follow-up.

### 2.2. Medical Care

In our hospital, eligible comatose patients after resuscitation from CPR and massive cerebral hemispheric infarction (MCHI) shortly underwent therapeutic hypothermia (TH) with a target core body temperature of 34-35°C for 24 hours, intravascularly or through the body surface, and were often provided with continuous infusion of midazolam or vecuronium.

### 2.3. EEG Data

Two experienced technologists performed bedside EEG 24–36 hours after coma (core body temperature 34-35°C). Moreover, we chose four patients (two in each group) randomly and retested the EEG-R during normothermia (core temperature > 36°C) and off sedation. Electrodes were placed according to the international 10–20 system, using 16 channels (Fp1, Fp2, F3, F4, F7, F8, C3, C4, T3, T4, T5, T6, P3, P4, O1, and O2) with Cz, A1, and A2 as references. All referential 16-channel EEG were recorded using a portable 32-channel digital EEG system (DAVINCI-SAM; Micromed, Mogliano Veneto, Italy). The Fpz was used as a ground. Fz was not performed on the scalp but was used to record stimulation information.

We selected the parts of the EEG recordings without prominent artifacts and tested EEG-R. EEG-R was tested using electrical stimulation, which was carried out with stimulator on the electromyography/evoked potential machine (Nicolet Viking IV, Nicolet, Madison, WI, USA). We applied somatosensory stimuli on the left or right median nerves of the upper extremity separately, just like the method of somatosensory evoked potentials (SEP). The stimulation was applied with 5 Hz square-wave pulses lasting 2 seconds followed by no less than 3 minutes of rest. This stimulation was repeated at least twice and bilaterally. To ensure that each subject received abundant stimulation, each patient was tested by SEP. The stimulus intensity was sufficient to produce a thumb twitch (0.5–1 cm) and was also recorded. We excluded patients without N9, N13, and P14, as the somatosensory stimulus could not be transmitted to the cerebrum.

The EEG-R was visually analyzed by two certified neurophysiologists (certified by Brain Injury Evaluation Quality Control Centre of China) separately. They could change the filters, signal gain, and montages. The EEG-R was classified as present (reactive) or absent (nonreactive). The inconsistent results would be solved through negotiation. The present EEG-R was defined as the presence of any clear change in amplitude or frequency following the application of quantifiable electrical stimuli.

### 2.4. Neurological Evaluation

Neurologic outcome was assessed at 3 months by one physician unaware of the clinical and EEG assessments through a phone interview. The outcomes were described according to the Glasgow-Pittsburgh cerebral performance categories (CPC) for CPR patients or the modified Rankin scale (mRS) score for stroke patients. In CPC, 1 = good recovery, 2 = moderate disability, 3 = severe disability with dependency for daily-life activity, 4 = vegetative state, and 5 = death, and the outcome was dichotomized as good (CPC 1–3) or poor (CPC 4-5). In mRS, 0 = no symptoms, 1 = no significant disability despite symptoms, 2 = slight disability, 3 = moderate disability, 4 = moderately severe disability, 5 = severe disability, and 6 = death. Likewise, the mRS score of 0–4 represented a good outcome, while 5-6 represented a poor outcome.

### 2.5. Statistical Analysis

SPSS statistical software, version 19.0 (SPSS Institute, Inc., Chicago, IL, USA), was used for all statistical analyses. We performed two-tailed *t*-tests for normally distributed continuous variables and chi-squared tests for confirmatory variables. A Mann-Whitney *U* test was performed in cases in which the variable was not normally distributed. The sensitivity, specificity, positive predictive value (PPV), and negative predictive value (NPV) were calculated to identify the prognostic value of EEG-R to the quantifiable stimulation in neurological outcomes of patients. *P* values less than 0.05 were considered statistically significant.

## 3. Results

### 3.1. Patients

Over the one-year enrollment period, 14 patients who suffered from CPR and 11 patients who suffered from stroke were admitted to our hospital. Three patients were excluded from analysis due to myoclonus, the absence of bilateral N9, or severe neurologic deficits. Thus, 22 patients were enrolled in this study, of which 12 patients were post-CPR and 10 patients had stroke. The leading reason of CPR was cardiac causes (*n* = 7), followed by anesthetic accident (*n* = 4) and pulmonary causes (*n* = 1). In the stroke group, 9 patients suffered from cerebral infarction (6 MCHI) and 1 patient suffered from intracerebral haemorrhage.

Of all the comatose patients, including those with CPR and stroke, 13 cases had good outcome and 9 cases had poor outcome. Among the 12 patients after CPR, 2 were VS and 3 were dead. The outcomes of 10 patients after stroke were as follows: 6 had good outcome, 2 suffered from severe disability, and 2 died. There was no statistical significance between the good and poor outcome groups.

These characteristics and clinical data are detailed in Table 1.

### 3.2. EEG-R and Outcomes

All patients were bilaterally tested, except for 1 patient with local impairment in the left upper extremity. The concordance rate of the two neurophysiologists was 90.9%. Two patients (patient 2 in CPR group and patient 6 in stroke patient) required negotiation to determine the final results. The repeated outcomes were coincident with the former. There were no differences in lateral and repeated tests. Moreover, there were no obvious artifacts when performing electrical stimulation.

Of the 12 patients after CPR, 7 cases were categorized as reactive and 5 were categorized as nonreactive. Among the 7 patients with EEG-R, 6 had good outcomes (PPV, 85.7%). Four of the 5 patients without EEG-R had poor outcomes (NPV, 80.0%). Furthermore, the sensitivity and specificity were 85.7% and 80.0%, respectively. The prognostic accuracy was good.

For 10 patients with stroke, 7 cases were categorized as reactive and 3 cases were categorized as nonreactive. Among the 7 patients with EEG-R, 6 had good outcomes (PPV, 85.7%). Additionally, all patients without EEG-R had poor outcomes (NPV, 100.0%). These data implied that the EEG-R also had strong prognostic value, with 100.0% sensitivity and 75.0% specificity.

For all the enrolled comatose patients, 13 cases had good outcome (12 present EEG-R) and 9 cases had poor outcome (7 absent EEG-R). The presence of EEG-R was a good positive factor for the prognosis of good outcome, with 92.3% sensitivity, 77.7% specificity, 85.7% PPV, and 87.5% NPV.

We retested EEG-R on 4 patients (patients 6 and 9 in the CPR group and patients 5 and 8 in the stroke group) 2-3 days after TH and withdrawal of sedative agents. All the outcomes of EEG-R were the same but with a higher degree of stimulus intensity.

**Figure 1:**
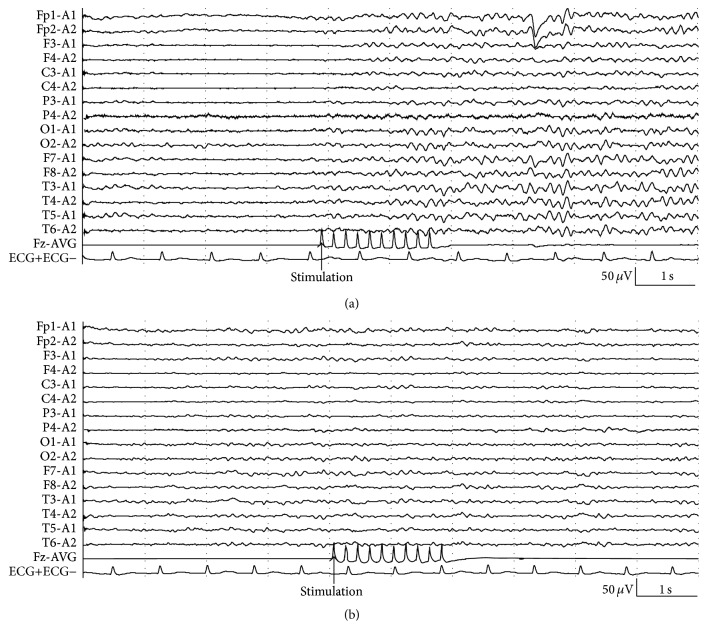
EEG-R to quantifiable electrical stimulation in comatose patients. (a) Present reactivity. (b) Absent reactivity. Fz was used to record stimulation information. The stimulation was applied with 5 Hz square-wave pulses lasting 2 seconds. The arrow represented the onset of stimulation.

The reactive and nonreactive EEG background to quantifiable electrical stimuli are shown in Figures 1(a) and 1(b). The prognostic values of EEG-R are shown in Table 2.

## 4. Discussion

EEG-R is a good positive factor for the prognosis of outcome in comatose patients. In this study, we first employed quantified electrical stimulation to predict the outcome of comatose patients in CPR or stroke. We found that quantified stimulation with electrical square-wave pulses had high sensitivity and specificity. Moreover, we did not find differences in lateral and repeated tests, suggesting that this new stimulation method was stable in clinical practice. Our study indicates that electrical stimulation may be a valuable method to predict outcome in comatose patients after CPR or stroke.

EEG reflects the electrical activities of neurons [10]. Therefore whether electrical stimulation may influence the recording of EEG is the first question we confronted. Our study showed that there was no striking artifact related to electrical stimulation. The potential causes are as follows. We chose the median nerves of the upper extremity as the stimulation area, which were far from the head. Moreover, the duration of stimulation was only 2 seconds. Additionally, we defined the standard stimulus intensity as that sufficient to produce a thumb twitch (0.5–1 cm), not only ensuring abundant stimulation for each subject but also avoiding possible artifact by high stimulation.

Compared to previous studies on EEG-R, our results showed good accuracy [6, 14, 15]. This may have benefitted from our quantitative stimulation method. EEG reactivity in comatose patients is mainly assessed by the visual comparison of EEG segments before and after the time of stimulus administration. The external stimulation often includes applying auditory stimuli by calling the patients' names, somatosensory stimuli by applying pressure to the nail bed, and visual stimuli by passive eye opening or light [16]. These stimulations are difficult to standardize in clinical practice and the intensity and duration of stimulation may differ by different persons, which can be avoided by quantitative stimulation. In a recent paper, thermal stimuli were quantified, and EEG-R induced by thermal stimulation (42 ± 2°C) showed good predictive accuracy in patients in VS or MCS, with high sensitivity (90%) and high specificity (81.8%) [13]. However, this method may not be suitable for comatose patients. First, erroneous EEG-R might be caused by artifacts when putting a plastic bag containing warm water on the patient's body surface. Second, this degree of temperature might not warrant the intensity of stimulation in comatose patients. Moreover, a higher temperature might result in damage to skin. Therefore, we employed quantified electrical stimulation and demonstrated that this quantitative stimulation method improved the accuracy for outcome prediction in comatose patients.

The prognostic value of EEG-R in comatose patients was first noted by Fishgold and later by Synek and Young [11, 16, 17]. EEG-R after external stimuli represents the neural activity along the afferent somatosensory pathways through the ascending reticular activating system (ARAS) to the cortex [8]. Additionally, reactivity is a function of the robust ARAS, it is analogous to behavioral arousal, and it can reflect the temporal synchronization of cortical pyramidal neurons [10]. Thus, EEG-R is an important factor that represents the function of the ARDS and cortex, which are important for human cognition and conscious awareness. Furthermore, functional magnetic resonance imaging (fMRI) has found that the brainstem, thalamus, primary and secondary somatosensory cortices, anterior cingulate, insula, prefrontal and inferior parietal cortices, and cerebellum can be activated by thermal stimuli [18, 19]. These findings further contribute to the underlying mechanism. However, compared with painful and thermal sensations, electrical sensation is conducted through a different neural transduction pathway [20]. Other studies are warranted to confirm the prognostic value of this new electrical stimulation method.

In our study, EEG was recorded 24–36 hours after coma, which was similar to previous studies. It is most accurate for predicting outcome within 48 hours [21–24]. Moreover, performing EEG test too early (within 24 hours) could even decrease the accuracy of prediction. Although previous studies have confirmed that mild hypothermia and a low dose of midazolam or propofol did not influence the prognostic value of the EEG pattern [21, 24], the confounding effects of sedation and TH on EEG-R still need further confirmation. In addition, sedative agents are inevitably used in some comatose patients, even without TH. For that reason, we retested EEG-R in 4 patients (2 CPR and 2 MCHI) during normothermia and off sedation. Our results showed that the degree of stimulus intensity was higher during TH with sedation. The possible reasons are listed as follows. Midazolam could decrease neuronal excitability by increasing the efficiency of g-amino butyric acid (GABA) receptors [25]. GABA is a type of neurotransmitter acting on chloride channels which would result in hyperpolarization and therefore decreased excitability [26]. In addition to increasing GABA, propofol can also inhibit N-methyl-D-aspartate (NMDA) receptor [27]. Moreover, TH can decrease metabolism, resulting in low excitability [28]. According to our results, the confounding effects caused by TH and the low-dose use of sedation drugs might be resolved by increasing stimulus intensity.

We also found that the outcome was different between CPR and MHCI (poor outcome: 41.6% versus 50.0%), which was similar to previous studies [29, 30]. The underlying reason might be the fact that the mechanisms involved in impairment in the cortex and ARDS are different. However, the difference did not reach statistical significance, possibly due to the small sample size in the current study.

Additionally, the mean age of good outcome was different between CPR and MCHI (43.9 versus 68.7). In MCHI patients, old patients had a lower risk of cerebral hernia caused by edema due to cerebral atrophy [31]. In the CPR group, 3 of 7 patients with good outcome experienced anesthetic accident without past pulmonary or cardiac diseases. Therefore, the role of age in our study requires further confirmation.

Although EEG-R was accurately predicted in comatose patients, a single assessment of EEG-R might not be sufficient for family counseling and end-life decisions. The background of EEG itself provides useful information [21, 24, 32, 33]. The prognostic significance in comatose patients has been detected by prior studies. A benign pattern, such as dominant theta activities, even with nonreactivity, does not certainly imply a bad prognosis [6]. Likewise, taking stimulus-induced rhythmic, periodic, or ictal discharges (SIRPIDs) as an example, the presence of EEG-R might also indicate an unfavorable prognostic outcome [34]. Therefore, combination of the EEG pattern with other neurophysiological tests might ensure the predictive outcome.

The limitations of this study are as follows. Our study sample was small. Thus, a large prospective trial is warranted to confirm the prognostic value of the quantitative electrical stimulation in comatose patients. We also acknowledged the heterogeneous population, which would make the subcategories much smaller yet. Moreover, we did not directly compare electrical stimulation with pain. Additionally, many studies have focused on the quantitative analysis of EEG features, which can detect subtle changes and increase objectivity [35, 36]. We will explore the value of quantified stimulation combined with quantitative analysis, such as temporal brain symmetry index, relative entropy, and Kolmogorov-Smirnov test.

## 5. Conclusions

Here, we report a new quantified method through electrical stimulation for EEG-R testing. Our results show that EEG-R through electrical stimulation is a significant predictive factor of clinical outcome in comatose patients after CPR or stroke. Further studies are warranted to confirm and refine this method.

## Figures and Tables

**Table 1:** Characteristics and outcomes of comatose patients.

Number		GCS	Sex	Age (y)	EEG-R	Score	Outcome
CPR	CPR causes					CPC	

1	Cardiac causes	7	M	51	Y	2	Good
2	Anesthetic accident	6	M	28	Y	2	Good
3	Cardiac causes	5	M	37	N	5	Poor
4	Cardiac causes	6	M	36	Y	2	Good
5	Anesthetic accident	7	M	26	N	1	Good
6	Cardiac causes	5	M	78	N	4	Poor
6^*∗*^	Cardiac causes	5	M	78	N	4	Poor
7	Anesthetic accident	6	M	39	Y	1	Good
8	Cardiac causes	6	M	77	Y	5	Poor
9	Anesthetic accident	5	F	78	Y	3	Good
9^*∗*^	Anesthetic accident	5	F	78	Y	3	Good
10	Pulmonary causes	7	M	49	Y	3	Good
11	Cardiac causes	6	M	58	N	4	Poor
12	Cardiac causes	4	M	36	N	5	Poor

Stroke	Type of stroke					mRS	

1	Hypodensity < 67% MCA territory	7	F	78	Y	4	Good
2	MCHI	8	F	62	N	6	Poor
3	Intracerebral haemorrhage	6	M	58	Y	4	Good
4	MCHI	6	F	66	Y	4	Good
5	MCHI	7	M	59	N	5	Poor
5^*∗*^	MCHI	7	M	59	N	5	Poor
6	MCHI	5	F	68	Y	3	Good
7	Hypodensity < 67% MCA territory	6	M	57	Y	5	Poor
8	MCHI	7	F	72	Y	4	Good
8^*∗*^	MCHI	7	F	72	Y	4	Good
9	MCHI	8	M	62	N	6	Poor
10	Hypodensity < 67% MCA territory	6	M	67	Y	3	Good

CPR, cardiopulmonary resuscitation; GCS, Glasgow Coma Scale; MCHI, massive cerebral hemispheric infarction; MCA, middle cerebral artery; F, female; M, male; Y, yes; N, no; CPC, Glasgow-Pittsburgh cerebral performance categories; mRS, modified Rankin scale. ^*∗*^The EEG-R was retested 2-3 days after TH and withdrawal of sedative agents.

**Table 2:** Prognostic value of EEG-R to quantifiable electrical stimuli for outcome at 3-month follow-up.

	Poor (*n*)	Good (*n*)	Se (%)	Sp (%)	PPV (%)	NPV (%)
CPR + stroke	9	13	92.3	77.7	85.7	87.5
CPR	5	7	85.7	80.0	85.7	80.0
Stroke	4	6	100.0	75.0	85.7	100.0

Se, sensitivity; Sp, specificity; PPV, positive predictive value; NPV, negative predictive value.
